# Public Stigma of COVID-19 and Its Correlates in the General Population of China

**DOI:** 10.3390/ijerph182111718

**Published:** 2021-11-08

**Authors:** Tian-Ming Zhang, Qi Fang, Hao Yao, Mao-Sheng Ran

**Affiliations:** 1 Department of Social Work, Shanghai University, Shanghai 200444, China; tianming_1989@hotmail.com; 2 School of Social Development and Public Policy, Fudan University, Shanghai 200433, China; 3 Shanghai Clinical Research Center for Mental Health, Shanghai Key Laboratory of Psychotic Disorders, Shanghai Mental Health Center, Shanghai Jiao Tong University School of Medicine, Shanghai 200030, China; 4 Department of Social Work and Social Administration, University of Hong Kong, Hong Kong, China; msran@hku.hk

**Keywords:** public stigma, COVID-19, correlates, attribution theory, China

## Abstract

This study aimed to examine the profile of COVID-19-related public stigma and its correlates in the general population of China. A cross-sectional online survey was conducted in China from 7 May to 25 May in 2020. A total of 1212 participants from the general population completed the survey measuring their stigmatizing attitudes towards COVID-19, as well as knowledge and causal attributions of COVID-19. Univariate and multivariate analyses were performed to examine the correlates of COVID-19-related public stigma. A total of 31.8% of participants endorsed stigmatization towards people with COVID-19. Those who were of older age (t = −3.97, *p* < 0.001), married (F = 3.04, *p* < 0.05), had a lower level of education (F = 8.11, *p* < 0.001), and a serious psychological response (F = 3.76, *p* < 0.05) reported significantly higher scores of public stigma. Dangerousness (*B* = 0.047, *p* < 0.001), fear (*B* = 0.059, *p* < 0.001), anger (*B* = 0.038, *p* < 0.01), and responsibility (*B* = 0.041, *p* < 0.001) were positively associated with public stigma. This study shows that public stigma related to COVID-19 is prevalent in the general population of China. Actions against public stigma need to contain the spread of misinformation about COVID-19, alter inappropriate attributions, alleviate unfavorable reactions, and provide psychosocial support for the public.

## 1. Introduction

Since the beginning of 2020, the pandemic of coronavirus disease 2019 (COVID-19) has swept the globe, with more than 248 million cases diagnosed till now [1]. The pandemic of COVID-19 not only jeopardizes the physical health of those infected with severe acute respiratory syndrome coronavirus 2 (SARS-CoV-2), but it also arouses a new wave of public stigmatization in the general population towards them [2]. In the context of health, public stigmatization is contextualized as a process of labeling, stereotyping, and discriminating against people with a certain disease [3]. It is also not uncommon that, as the outbreaks of newly emerging infectious diseases escalate, disease-related public stigmatization often follows inside and beyond the hot zones [4]. Recent examples of public stigmatization during infectious disease outbreaks include severe acute respiratory syndrome (SARS) [5], H1N1 influenza [6], Ebola [7], and Zika [8].

The ramifications of stigmatization towards COVID-19 patients might be manifold. First, it may hinder COVID-19 patients’ help-seeking behaviors, leading to unnecessary delays in diagnosis and treatment and promoting continued infection transmission of COVID-19. Second, in addition to COVID-19 per se, COVID-19-related public stigmatization may increase COVID-19 patients’ psychological burdens and therefore worsen their clinical outcomes. Third, even for those patients who survived, stigma may continue to be attached to them, resulting in social exclusion and ostracism and eventually compromising their physical, emotional, and mental health. Moreover, given sociodemographic differences (e.g., based on race/ethnicity, gender, and age) of COVID-19 incidence and mortality [9,10,11,12,13,14,15,16], public stigmatization towards COVID-19 patients can exacerbate the already pervasive “us vs. them” mentality in society, and undermine social cohesion and global solidarity which matters even more during the crisis of COVID-19. 

Given the severe epidemic situation of COVID-19 across the globe, public stigma of COVID-19 is more likely to be our threat than the disease itself [17]. Misconceptions and lack of knowledge about COVID-19, along with high fear levels of COVID-19, have been confirmed to be associated with more stigmatizing attitudes towards people associated with the context of COVID-19, such as healthcare providers and Asians in the United States [18,19,20]. A national online survey in Bangladesh reported that stigmatizing attitudes related to COVID-19 in the general population was significantly associated with marital status, educational level, living conditions, and perception of risk [21]. Research on public stigma posited that individuals’ perceptions of stigmatized conditions were associated with their cognitive attributions and affective responses [22]. According to Weiner’s attribution theory, if individuals are perceived to be capable of controlling the occurrence of their diseases and to be responsible for their own infections, they are more likely to be stigmatized [23]. In addition to causal attributions about controllability and responsibility, individuals’ fearful and irritated responses are also linked with stigmatization [24]. Such correlations have been found in numerous studies about public stigmatization towards mental illness, HIV/AID, and disabilities [25,26,27]. Within the framework of attribution theory and prior empirical studies, we proposed that public stigma towards people with COVID-19 would be linked with different attributions and unfavorably emotional responses. 

Given that the dual pandemic of COVID-19 and COVID-19-related public stigma still escalates worldwide, it is of great importance to investigate and to address COVID-19-related public stigma in the general population. Understanding its profile and correlates can help to develop targeted interventions to reduce it, as well as its individual- and societal-level ramifications. However, such studies are still scarce around the world. Therefore, the present study aimed to examine the profile of COVID-19-related public stigma and its correlates in the general population of China through the lens of attribution theory. 

## 2. Materials and Method

### 2.1. Participants and Procedure

A large convenience sample was recruited from the general population in China from 7 May to 25 May in 2020 during the first wave of the COVID-19 pandemic in China. An online questionnaire was created using Qualtrics, a commonly used online survey tool, and distributed through social media applications widely used in China (e.g., Weibo, Douban, and WeChat). The informed consent form was presented on the first page of the online questionnaire. Before filling out the rest of the questionnaire, the informed consent was obtained by clicking the AGREE button on that page. Individuals who were aged 18 years or older and living in Mainland China were eligible to participate in this study. Those who were infected with COVID-19, did not complete the questionnaires, or whose IP addresses were not in Mainland China were excluded. The final sample of the present study consisted of 1212 participants, which met the WHO requirement about the sample size (N = 1000) of COVID-19-related online surveys [28]. Ethical approval was obtained from the Ethics Committee of Shanghai Mental Health Center, Shanghai Jiao Tong University (Reference Number:2020-33). 

### 2.2. Measurements

Public Stigma of COVID-19 Scale: It was modified from an existing scale of stigma attached to mental illness [29]. The original scale includes two dimensions, with 12 items measuring public stigma of mental illness and 9 items measuring acceptance of mental illness. In the present study, we only extracted the dimension of public stigma and altered the related disease from mental illness to COVID-19. Given people with COVID-19 would be forcibly quarantined in China, we removed one item: *“People with COVID-19 should be quarantined”*. Therefore, the final version of the Public Stigma of COVID-19 Scale consisted of 11 items, such as *“People with COVID-19 are revolting”*. Items were rated on a 5-point Likert scale (1 = strongly disagree, 5 = strongly agree), with higher scores indicating more public stigma. The Public Stigma of COVID-19 Scale had sound internal consistency (Cronbach’s alpha = 0.83).

Knowledge about COVID-19: A 20-item questionnaire was developed to measure participants’ familiarity with key facts about COVID-19. The items were extracted from the *Diagnosis and Treatment Protocol for Novel Coronavirus Pneumonia* (Trial Version 7) and the *Prevention Protocol for Novel Coronavirus Pneumonia* (Version 6) released by China’s National Health Commission, such as “*The new coronavirus can continue to spread from person to person.*” Items were rated on a 5-point Likert scale (1 = strongly unfamiliar, 5 = strongly familiar), with higher scores indicating more familiarity with key facts about COVID-19. 

Attributions of COVID-19: We developed a 6-item questionnaire to measure participants’ causal attributions of COVID-19, i.e., dangerousness, fear, anger, blame, controllability, and responsibility. The three items measuring dangerousness, fear, and anger were extracted from Corrigan and his colleagues’ work on attributions of mental illness [30]. The other three items measuring blame, controllability, and responsibility were extracted from a study about public stigma of infectious diseases [26]. For instance, the item “*People infected with COVID-19 are dangerous*” was used to measure the attribution about dangerousness. Participants were asked to rate their agreement with each item on a 9-point Likert scale (1 = not at all, 9 = very much), with higher scores indicating more agreement. 

Sociodemographic characteristics and other general information: We used a self-administered questionnaire to collect participants’ sociodemographic characteristics and other general information, including gender, age (<=30 years, >30 years), marital status (single, married, others), and education level (≤high school, =college, ≥graduate), employment status (employment, unemployment, retired, student), monthly income (RMB), whether to work on the health frontline during the outbreak of COVID-19 (yes or no), psychological response to the pandemic (mild, moderate, severe).

### 2.3. Statistical Analysis

Stata/SE 16.0 was used for data analysis. Descriptive analysis was performed to summarize the sociodemographic characteristics by presenting percentages, means, and standard deviations. We used the independent-samples *t*-test and analysis of variance (ANOVA) to compare the scores of public stigma by categories of various sociodemographic characteristics. As for the Public Stigma of COVID-19 Scale, we calculated the means and standard deviations for each item. In addition, agreement or strong agreement with one certain item of the Public Stigma of COVID-19 Scale was considered as endorsement. In terms of total public stigma, respondents who reported average scores greater than 3 were treated as endorsing public stigma. We presented the prevalence of endorsement of each item and the total public stigma. A correlation matrix was used to examine the correlations between public stigma of COVID-19 and other continuous variables including knowledge about COVID-19, causal attributions (i.e., dangerousness, fear, blame, anger, controllability, and responsibility), and willingness for help-seeking. In addition, a stepwise linear regression with different blocks was applied to examine the correlates of public stigma. Variables significantly associated with public stigma in univariate analyses were entered into three models of multivariate linear regression. Model 1 included demographic factors, model 2 involved general information related to COVID-19, and model 3 was related to causal attributions of COVID-19. *P*-values of < 0.05 were deemed to be significant in this study.

## 3. Results

### 3.1. Sociodemographic Characteristics

Table 1 displays the sociodemographic profile of 1212 participants, as well as the scores of public stigma by categories of various sociodemographic characteristics. The majority of the participants were female (73.27%) and were less than or equal to 30 years old (73.27%). Over half of the participants were single (65.10%) and had a college degree (68.65%). A small percentage of the participants (12.16%) worked on the health frontline of the COVID-19 pandemic. Most of the participants (64.36%) self-reported a severe psychological response to the pandemic. Participants’ age group (t = −3.97, *p* < 0.001), marital status (F = 3.04, *p* < 0.05), level of education (F = 8.11, *p* < 0.001), and psychological response to the pandemic (F = 3.76, *p* < 0.05) were significantly associated with public stigma. Participants who were of younger age, single status, had higher levels of education and a mild psychological response reported lower scores of public stigma. There were no significant differences on scores of public stigma by participants’ gender, employment status, monthly income, and health frontline status. 

### 3.2. Endorsement of Public Stigma 

The means, standard deviations, and prevalence of endorsement of each item in the Public Stigma of COVID-19 Scale are shown in Table 2. Three items were endorsed by more than 50% of the entire sample, that is 1) “*I will try my best to keep a distance from people with COVID-19*” (79.04%), 2) “*I am afraid of being alone with people with COVID-19*” (62.13%), and 3) “*I am worried that people with COVID-19 will cause harm to others*” (50.66%). Meanwhile, the mean scores of these three items ranked the highest among all items (1 = 4.10, 2 = 3.65, and 3 = 3.34, respectively). Overall, the mean total score of the Public Stigma of COVID-19 Scale was 2.68, with 31.80% participants endorsing COVID-19-related public stigma. 

### 3.3. Relationships between Public Stigma and Continuous Variables

Table 3 provides a correlation matrix of public stigma and other continuous variables. Knowledge about COVID-19 was negatively associated with public stigma (r = −0.057, *p* < 0.001), whereas causal attributions about dangerousness (r = 0.364, *p* < 0.001), fear (r = 0.403, *p* < 0.001), blame (r = 0.247, *p* = 0.001), anger (r = 0.252, *p* < 0.001), controllability (r = 0.195, *p* < 0.001), responsibility (r = 0.308, *p* < 0.001), and willingness of help-seeking (r = 0.063, *p* < 0.05) were positively associated with public stigma.

### 3.4. Factors Associated with Public Stigma Using Multiphase Linear Regression

To identify the correlates of COVID-19-related public stigma, we performed a stepwise linear regression with three blocks, using the variables significantly associated with public stigma in univariate analyses. Demographic factors, general information related to COVID-19 (i.e., psychological reaction, knowledge, willingness for help-seeking), and causal attributions of COVID-19 (i.e., dangerousness, fear, blame, anger, controllability, and responsibility) were entered into the linear regression model sequentially (Table 4). 

In Model 1, the age group and education level were significantly associated with public stigma. Compared with participants of younger age and with higher levels of education, those of older age (*B* = 0.182, *p* < 0.01) and with lower levels of education (*B* = −0.202, *p* < 0.01) scored higher on public stigma. In Model 2, it was shown that compared with participants with a severe psychological response to the pandemic of COVID-19, those with a mild response reported a significant lower score of public stigma (*B* = −0.172, *p* < 0.01). In Model 3, the significant relationship between psychological response and public stigma disappeared after adding causal attributions of COVID-19. Model 3 showed that the more dangerous (*B* = 0.047, *p* < 0.001), fear (*B* = 0.059, *p* < 0.001), and angry (*B* = 0.038, *p* < 0.01) participants felt towards the people with COVID-19, the more public stigma they would endorse. Moreover, the more participants considered that people infected with COVID-19 was their own responsibility (*B* = 0.041, *p* < 0.001), the higher levels of public stigma they had. After entering all covariates, Model 3 was capable of explaining 25.4% of the variances in the public stigma towards people with COVID-19. 

## 4. Discussion

The present study examined public stigma associated with COVID-19 and its correlates in the general Chinese population during the disease’s epidemic. Our findings suggest that a high level of stigma towards people with COVID-19 was endorsed by the general population in China. In addition, an association between knowledge about COVID-19 and public stigma was found to be significant. Premised on an attribution model, the study findings contribute to understanding relationships between COVID-19 related public stigma and various attributors (dangerousness, fear, anger, and responsibility). 

Previous studies suggested that the general population commonly endorsed avoidant attitudes toward people with infectious diseases similar to COVID-19, such as SARS, Ebola, during the disease outbreak [31,32]. The current study also found that the proportion of participants holding such shunning notion was elevated. Nearly 80% of the participants preferred to keep social distancing with people infected with COVID-19, and more than half of the participants showed worries and fear when they came into contact with people with COVID-19. In term of affective aspects, however, only a few participants showed some tendency towards revolting or annoying attitudes. This finding might suggest that public perceptions of people with COVID-19 were contradictory. Given the infectiousness and mortality of COVID-19, it is not surprising that the general public approve of evading people suffering from COVID-19. Additionally, a strict quarantine policy in China during the pandemic might have increased the likelihood of people’s stigmatizing attitudes. A previous study conducted in Wuhan showed that those who were more familiar with quarantined cases were more likely to perceive higher levels of stigma [33].

The sociodemographic factors shown to be correlated with public stigma included age, marital status, education level, and psychological reaction. Similar to relevant studies on SARS [34,35], our study also found that younger participants were less likely to endorse stigmatizing attitudes. Given that those who were older were at greater risk when they had COVID-19 [36], it might be reasonable for them to show greater tendency of negative prejudice to people with COVID-19. The current study showed that those who were married were more inclined to hold stigmatizing ideas, which is in line with previous related study pertaining to SARS [32]. In terms of education level, prior research showed inconsistent findings. One study found no significate correlation between education level and discrimination on SARS [35]. In this study, we found participants with a higher education level were more likely to exhibit prejudice towards people with COVID-19. In addition, those who had a severe psychological reaction to the pandemic reported higher public stigma. The COVID-19 pandemic might exert a threat to the mental status of the general public and cause psychological distress, such as anxiety and depression [37,38,39,40]. Meanwhile, these adverse affective reactions might influence the public’s attitudes towards people with COVID-19.

The negative correlation between public stigma towards people with COVID-19 and knowledge, as shown in this study, has been repeatedly verified in other similar stigmatizing conditions, including infectious disease and mental disorders [41,42]. The findings of this study suggested that misconceptions of COVID-19 were correlated with stigmatizing notions. Prior studies pertaining to stigmatization also indicate that knowledge is one of the essential factors reducing prejudice towards the stigmatized group [43,44]. For the future, with the outbreak of newly emerging infectious disease, accurate information needs to be disseminated to reduce public misunderstanding, thereby reducing social stigma. Interestingly, after adjusting for other factors in the regression model of this study, however, the significant correlation between knowledge and public stigma disappeared. It might indicate that the role of knowledge on generating public attitudes was negligible given that COVID-19 was a novel infectious illness, and which has been confirmed in a previous study [26]. The extent to which knowledge diminishes stigmatizing ideas, thus, warrants further examination.

Plenty of prior studies, based on attribution theory, have indicated that controllability is significantly correlated with stigmatizing ideas [24,45,46,47]. However, this correlation was not shown in this study. A possible explanation may be related to the feature of COVID-19. Although COVID-19 is a highly contagious disease, people can recover by medical treatment and the disease does not pose momentous outcomes to others as long as the infected individuals receive timely treatment. A previous study compared public stigma amongst three infectious illnesses and concluded that disease attribution and stigma were subordinated to characteristics of the disease itself. Specifically, compared with SARS, the general population believed infection of HIV/AIDS to be more controllable by the person, as a result, they held more stigmatization towards people with HIV/AIDS [26]. We found that the general population who attributed more personal responsibility to people having COVID-19 were inclined to endorse more stigma. In line with previous studies related to stigma, the current study also confirmed that public stigma were significantly connected to the general populations’ effective response to the people infected with COVID-19 [18]. A prior study indicating that great danger appraisal predicted more stigmatizations could support the results of this study [48]. For instance, those who felt more dangerous and expressed more fear and anger were more likely to perceive stigmatizing ideas. 

Several limitations in this study should be acknowledged. First, since the data was collected during the outbreak of COVID-19, we utilized an online survey method which is feasible and safe considering the transmission of coronavirus. As a result, those who had limited access to the internet, such as elderly people, were less likely to participate in our study. Another feature of the sample distribution in this study was that the majority of respondents were female (73.27%). This might be explained by gender difference [49], as several previous studies collecting data during the COVID-19 outbreak by using an online survey also reported a similar gender discrepancy in the sample distribution [50,51]. Given the time of assessment of this study, it may also have an effect. The high level of public stigma might relate to the uncertainty about the virus and the lack of scientific knowledge at the outbreak of COVID-19. However, this round of data might still be used as a baseline to provide a reference for a long-term follow up survey of the public stigma related COVID-19. In addition, a cross-sectional design cannot demonstrate the cause-and-effect relationships among knowledge, attribution factors, and public stigma. Thus, a longitudinal design should be considered in future studies to elucidate the effect of attribution on public stigma and to examine the attribution model in COVID-19. With respect to measurements, the scale measuring public stigma was modified from an instrument used in the issue attached to mental illness. Given the possible divergence of the stigma mechanism between mental illness and COVID-19, it might not capture the full profile of the COVID-19-related stigma. Even though the reliability of the instruments for public stigma in this study is acceptable, it would be better if we could use a more standardized measurement in a future study for comparison across various research studies. Finally, several other factors which are not contained in the present study might explain attitudes towards COVID-19 in the general population as well. For instance, the media reports of untrue information about disease transmission and coverage of infected people with the disease label might increase the likelihood of stigmatization ideas in the general population [52].

Despite these aforementioned limitations, our findings support the need to design public stigma reduction programs attached to COVID-19 and to shed light on the significance of health policy responses at the outbreaks of newly emerging infectious diseases. The current study demonstrates the association between lack of knowledge and endorsement of stigmatizing notions among the general population, which implies the necessity of disseminating accurate information of COVID-19 to diminish misunderstanding. In addition, our study indicates stigma to be related to attributions of the disease. The policies targeted to reduce stigmatizing attitudes, therefore, should focus on guiding the public to have appropriate attributions of the COVID-19 infection. In the long run, anti-stigmatization should be a focus of public health policies to make the public hold a more accepting attitude towards infectious disease (e.g., COVID-19, etc.) and infected people. 

## 5. Conclusions

Our study identified the profile of public stigma during the outbreak of COVID-19 in China, which might warrant the necessity for researchers and policy makers to tailor and implement anti-stigma interventions to challenge bias and prejudice for people facing a sudden outbreak of infectious disease like COVID-19. Given the role of attributions influencing public attitudes towards people with COVID-19, it is not only essential to design health communication programs to contain the spread of misinformation about COVID-19, but also to develop strategies to alter inappropriate causal attributions of COVID-19 in the general population. On account of the significant relationships between emotional response (e.g., fear, anger) and public stigma, more attention should be placed on alleviating these unfavorable reactions and providing psychosocial support for individuals trapped in emotional distress due to the pandemic. Currently, the COVID-19 pandemic is still continuing around the world. Nevertheless, the social stigmatization might be even more prolonged and extend to recovered patients and healthcare professionals according to the preceding stigma studies related to SARS and Ebola [31,34,53]. Therefore, stigmatization towards COVID-19 should be understood and addressed in the context of combatting infectious diseases.

## Figures and Tables

**Table 1:** Sociodemographic characteristics and their differences in public stigma score.

Characteristic	N	%	Public Stigma Score		Test Statistic	*p*
			Mean	SD		
**Gender**					*t* = 0.22	0.827
Male	324	26.73	2.68	0.64		
Female	888	73.27	2.67	0.63		
**Age(years)**					*t* = −3.97	<0.001
<=30	888	73.27	2.63	0.63		
>30	324	26.73	2.74	0.63		
**Marital status**					*F* = 3.04	0.048
Single	789	65.10	2.65	0.63		
Married	325	26.82	2.75	0.67		
Others	98	8.09	2.66	0.57		
**Education level**					*F* = 8.11	<0.001
<=High school diploma or below	115	9.49	2.85	0.66		
=Bachelor’s degree	823	68.65	2.69	0.63		
>=Graduate degree	265	21.86	2.57	0.60		
**Employment status**					*F* = 2.19	0.087
Employed	451	37.21	2.71	0.65		
Unemployed	91	7.51	2.75	0.64		
Retired	41	3.38	2.80	0.61		
Student	629	51.90	2.63	0.62		
**Monthly Income (RMB)**					*F* = 1.29	0.274
<4000	785	64.77	2.67	0.64		
4000–8000	275	22.69	2.72	0.61		
>8000	152	12.54	2.62	0.65		
**Working on the frontline**					*t* = 0.37	0.710
Yes	208	12.16	2.69	0.65		
No	1004	82.84	2.67	0.63		
**Psychological response to the pandemic**					*F* = 3.76	0.024
Mild	140	11.55	2.55	0.63		
Moderate	292	24.09	2.66	0.62		
Severe	780	64.36	2.70	0.66		

Abbreviations: SD: standard deviation, RMB: Ren Min Bi, the official currency of China.

**Table 2:** Mean scores and percentages of endorsement of public stigma of COVID-19.

Items	Mean	SD	Percentage of Endorsement of Public Stigma of COVID-19 (%)
People with COVID-19 are revolting.	1.88	0.95	4.04
I am worried that people with COVID-19 will cause harm to others.	3.34	1.05	50.66
I will try my best to keep a distance from people with COVID-19.	4.10	0.94	79.04
People with COVID-19 are annoying.	1.85	0.95	4.70
People with COVID-19 are a burden to society.	1.81	0.94	5.28
When I know someone is infected with the new coronavirus, I will alienate him/her.	2.93	1.51	31.93
The actions of people with COVID-19 are infuriating.	2.11	1.04	8.42
People with COVID-19 will cause trouble to others.	2.45	1.10	17.94
I am afraid of being alone with people with COVID-19.	3.65	1.14	62.13
It is normal for people with COVID-19 to be discriminated against by others.	2.14	1.04	9.80
When encountering someone who has traveled to an epidemic area, it is best to avoid him/her.	3.17	1.11	43.73
Total scale	2.68	0.63	31.80

Abbreviations: SD: standard deviation.

**Table 3:** Correlations between public stigma of COVID-19 and other continuous variables.

	1	2	3	4	5	6	7	8	9
1 public stigma	1								
2 knowledge	−0.057 ***	1							
3 dangerousness	0.364 ***	0.141 ***	1						
4 fear	0.407 ***	0.093 **	0.720 ***	1					
5 blame	0.247 ***	−0.198 ***	0.104 ***	0.170 ***	1				
6 anger	0.252 ***	−0.179 ***	0.066 ***	0.143 ***	0.606 ***	1			
7 controllability	0.195 ***	−0.094 **	0.080 **	0.110 ***	0.332 ***	0.360 ***	1		
8 responsibility	0.308 ***	−0.083 **	0.186 ***	0.204 ***	0.335 ***	0.379 ***	0.433 ***	1	
9 help-seeking	0.063 *	−0.219 ***	−0.059 *	−0.017	0.349 ***	0.301 ***	0.148 ***	0.159 ***	1

Notes: * *p* < 0.05, ** *p* < 0.01, *** *p* < 0.001.

**Table 4:** Linear regression models of public stigma of COVID-19.

	Model1	Model2	Model3
	*B* (95%CI)	*B* (95%CI)	*B* (95%CI)
**Age (years) (<=30=0)**	0.182 (1.058, 1.359) **	0.183 (1.060, 1.359) **	0.122 (1.012, 1.260) *
**Marital status(Single=0)**			
Married	−0.067 (0.826, 1.060)	−0.058 (0.833, 1.069)	−0.036 (0.865, 1.076)
Others	0.001 (0.877, 1.143)	0.009 (0.884, 1.152)	0.001 (0.891, 1.124)
**Education level (<=High school diploma = 0)**			
=Bachelor’s degree	−0.083 (0.803, 1.055)	−0.062 (0.820, 1.078)	−0.101 (0.801, 1.019)
>=Graduate degree	−0.202 (0.703, 0.949) **	−0.175 (0.721, 0.977) *	−0.176 (0.734, 0.958) **
**Psychological response to the pandemic (Severe = 0)**			
Moderate		−0.066 (0.860, 1.019)	0.022 (0.948, 1.102)
Mild		−0.172 (0.752, 0.943) **	−0.060 (0.852, 1.043)
**Knowledge of COVID-19**		−0.002 (0.995, 1.001)	−0.003 (0.994, 1.000)
**Willingness for help-seeking**		0.021 (0.994, 1.051)	−0.013 (0.962, 1.014)
**Dangerousness**			0.047 (1.027, 1.069) ***
**Fear**			0.059 (1.041, 1.082) ***
**Blame**			0.020 (0.998, 1.044)
**Anger**			0.038 (1.012, 1.066) **
**Controllability**			0.007 (0.992, 1.023)
**Responsibility**			0.041 (1.027, 1.058) ***
Summary statistics			
Adjusted *R*^2^	0.0173	0.0251	0.254

Notes: * *p* < 0.05, ** *p* < 0.01, *** *p* < 0.001.

## Data Availability

The data presented in this study are available on request from the corresponding author. The data are not publicly available dur to privacy.
